# Federating European REgistries for Stroke: Principles, Data Model and Future Perspectives for a Federated Analysis of Multiple Prospective Stroke Registries

**DOI:** 10.1111/ene.70715

**Published:** 2026-08-04

**Authors:** Alexander Salerno, Georges Melissargos, Mira Katan, Michael Knoflach, Stefan Kiechl, Joseph Harbison, Leonardo Renieri, Danilo Toni, Lina Palaiodimou, Georgios Tsivigoulis, Andreas Ktenidis, Theofilos Mailis, Yannis Ioannidis, Patrik Michel, Philippe Ryvlin, the FERES collaborators

**Affiliations:** ^1^ Stroke Center, Department of Clinical Neurosciences Lausanne University Hospital and University of Lausanne Lausanne Switzerland; ^2^ NeuroDigital@NeuroTech Lab, Department of Clinical Neurosciences Lausanne University Hospital and University of Lausanne Lausanne Switzerland; ^3^ Department of Neurology University Hospital Basel and University of Basel Basel Switzerland; ^4^ Department of Neurology Medical University of Innsbruck Innsbruck Austria; ^5^ Department of Medical Gerontology Trinity College Dublin Dublin 2 Ireland; ^6^ Interventional Neurovascular Unit Azienda Ospedaliero Universitaria Careggi Florence Italy; ^7^ Department of Human Neurosciences Sapienza University of Rome Rome Italy; ^8^ Second Department of Neurology “Attikon” University Hospital, School of Medicine, National and Kapodistrian University of Athens Athens Greece; ^9^ Athena Research & Innovation Center in Information Communication & Knowledge Technologies Athens Greece

**Keywords:** benchmarking, common data elements, confidentiality, data anonymization, federated learning, medical informatics applications, multicenter study as topic, quality of health care, registries, stroke

## Abstract

**Background:**

Acute ischemic stroke is a leading cause of death and disability. Despite strong evidence supporting reperfusion therapies and Stroke Unit care, access and quality of stroke services remain heterogeneous across Europe. Although national stroke registries provide valuable real‐world data, fragmentation, limited interoperability, and data protection constraints have restricted multinational analyses and benchmarking.

**Methods:**

The Federating European REgistries for Stroke (FERES) initiative establishes a GDPR‐compliant federated framework for secondary use of stroke registry data. FERES harmonizes heterogeneous datasets through a stroke‐specific Common Data Elements (CDE) model and performs analyses locally within each registry using the Medical Informatics Platform, sharing only aggregated, non‐identifiable outputs. To validate the framework, a predefined showcase analysis comparing anterior versus posterior circulation acute ischemic stroke was executed in both centralized and federated modes using an identical harmonized dataset.

**Results:**

FERES connected five national registries from Austria, Greece, Ireland, Italy, and Switzerland within a unified federated infrastructure. At the time of analysis, 149,772 patient events from two registries were accessible for federated querying, with three additional registries technically integrated and in advanced onboarding. The harmonized ontology comprised 945 standardized variables spanning the stroke care pathway. Federated execution reproduced centralized pooled‐data results across descriptive statistics, hypothesis testing, effect sizes, and multivariable regression models with only minimal numerical discrepancies.

**Conclusions:**

FERES demonstrates that large‐scale, multinational stroke research and benchmarking can be conducted in Europe using a privacy‐preserving federated approach, providing a scalable foundation for cross‐border real‐world evidence generation and quality improvement.

## Graphical abstract

The FERES initiative establishes a GDPR‐compliant federated framework for secondary use of stroke registry data across European countries. It harmonizes heterogeneous datasets through a stroke‐specific Common Data Elements (CDE) model and performs analyses locally within each registry sharing only aggregated, non‐identifiable outputs.

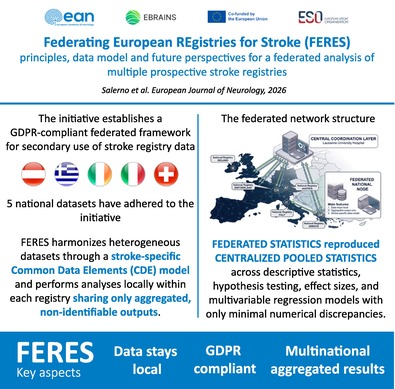

## 1 Introduction

Acute ischemic stroke remains one of the leading causes of mortality and long‐term disability worldwide [1, 2]. Over the past three decades, intravenous thrombolysis and endovascular thrombectomy have transformed acute management, with clear evidence that treatment benefit is highly time‐dependent [3, 4, 5]. Beyond reperfusion, organized multidisciplinary care in dedicated Stroke Units reduces mortality and disability and improves secondary prevention [6, 7, 8]. Together, rapid recognition, timely reperfusion, and structured post‐acute care form an integrated stroke survival chain essential to reducing the burden of stroke [9, 10, 11].

Despite robust evidence and clear guideline recommendations, access to evidence‐based stroke care remains highly heterogeneous worldwide and across Europe [12, 13, 14]. The Stroke Action Plan for Europe and surveys conducted by the European Stroke Organization have highlighted that substantial disparities persist in infrastructure, endovascular availability, Stroke Unit coverage, and implementation of secondary prevention strategies [15, 16]. A recent cross‐sectional analysis from the Registry of Stroke Care Quality (RES‐Q), encompassing over 218,000 ischemic stroke patients from 47 countries, demonstrated marked international variability in key quality indicators, with intravenous thrombolysis rates ranging from less than 1% to 52%, Stroke Unit or ICU admission rates from 12% to 100%, and door‐to‐needle times from 20 to 75 min [13]. Endovascular thrombectomy remains particularly underutilized: although 15%–20% of ischemic stroke patients may be eligible, adoption rates range from less than 1% to 7% across regions, and a European survey estimated that only 1.9% of all stroke patients undergo mechanical thrombectomy [17]. These gaps are compounded by geographic and socioeconomic disparities. A comprehensive meta‐analysis of over 5.2 million patients (the DARTS study) found that rural populations had significantly lower odds of receiving EVT compared with urban populations (OR 0.39, *p* < 0.001), and patients with low socioeconomic status faced similarly reduced access (OR 0.74, *p* < 0.001) [15]. Even within countries with universal tax‐funded healthcare, socioeconomic inequalities in thrombectomy rates persist [18]. A global survey across 89 countries identified cost, late presentation, and shortage of trained neurointerventionalists as the most common barriers to EVT delivery, with 22% of respondents reporting no available thrombectomy service [19]. Furthermore, a recent cost‐effectiveness analysis across 32 European countries demonstrated that mechanical thrombectomy is cost‐effective in virtually all settings, yet implementation remains far below optimal levels [20]. Consequently, many patients do not receive proven therapies within optimal time windows [21, 22, 23, 24]. Systematic evaluation of real‐world stroke care delivery across countries is therefore critical to inform policy and benchmark quality [25, 26].

Beyond benchmarking, large‐scale real‐world data complement randomized controlled trials (RCTs) [27, 28]. While RCTs establish efficacy under controlled conditions, they often include selected populations and are underpowered for rare subgroups or complex clinical scenarios. Pan‐European registry data enable assessment of treatment effectiveness in unselected populations, evaluation of external validity, and investigation of clinically relevant questions that are impractical for conventional trials [29, 30]. Examples include the management of posterior circulation strokes, isolated extracranial carotid artery occlusions, or other less frequent stroke subtypes where recruitment into randomized studies is challenging. They also allow analysis of modest treatment effects, rare stroke subtypes, long‐term outcomes, healthcare utilization, and system‐level interventions. Aggregation of high‐quality stroke data at scale is therefore essential to advance precision stroke care and optimize health system performance.

National and regional stroke registries are central to this effort. They support continuous quality monitoring, benchmarking, evaluation of organizational reforms, and validation of prognostic or therapeutic models in routine practice. However, many registries operate in isolation, and heterogeneity in data models, coding standards, and governance frameworks, combined with strict data protection regulations, limits cross‐border collaboration. As a result, European stroke research remains fragmented, and opportunities to generate robust, generalizable real‐world evidence at a continental scale are underexploited.

Traditional centralized research models based on pooling patient‐level datasets face increasing legal and logistical constraints, particularly under the General Data Protection Regulation (GDPR) [31, 32]. Cross‐border data transfer requires complex agreements and regulatory approvals, whereas analyses confined to single registries lack statistical power and geographic representativeness. This tension necessitates alternative analytical frameworks [33]. Understanding and addressing these disparities at a continental scale requires analytical approaches that can harness data from diverse healthcare systems while respecting regulatory constraints.

Federated data analysis offers a paradigm shift [33, 34, 35]. Instead of transferring patient‐level data to a central repository, standardized analytical workflows are deployed locally within each participating registry. Computations are performed behind institutional firewalls, and only aggregated, non‐identifiable results are shared [34, 35]. This model preserves data sovereignty, ensures regulatory compliance, and enables collaborative research at scale without compromising patient privacy [34].

The Federating European REgistries for Stroke (FERES) initiative was established to address these challenges. Built upon the Medical Informatics Platform (MIP) [36, 37, 38] developed within the Human Brain Project and further integrated into the EBRAINS research infrastructure [37], FERES provides a secure, privacy‐preserving environment for federated stroke analytics across Europe. The initiative harmonizes heterogeneous registry datasets into a common stroke‐specific data model covering the entire patient pathway and operates under a structured governance framework defining participation, access, and publication rules. By combining clinically meaningful harmonization with privacy‐by‐design technology, FERES aims to create a scalable and sustainable infrastructure for collaborative stroke research and quality improvement across Europe.

We describe the FERES framework and present a federated showcase analysis demonstrating feasibility and analytical reproducibility while maintaining strict local data control.

## 2 Methods

### 2.1 Ethical and Regulatory Framework for Federated Data Analysis

FERES was designed to comply with GDPR (Regulation (EU) 2016/679) and applicable national regulations. Analyses are executed locally at each participating institution (“local node”) on anonymized datasets that remain on‐site; no patient‐level data leave the local node. Only aggregated, non‐identifiable outputs are transmitted to the central coordination layer.

Under GDPR definitions, each participating registry or hospital acts as the “data controller”, retaining full responsibility for the purposes and means of processing its data, while the MIP operators act solely as “data processors”, providing the technical infrastructure without accessing or owning the data. These roles and responsibilities are formalized through federation agreements.

Participation required authorization for research reuse under local legal/ethical frameworks, with site‐specific approvals as applicable. Safeguards include role‐based access control, audit logging, encrypted communications, and suppression of outputs derived from < 10 individuals.

### 2.2 Identification and Inclusion of European Stroke Registries

For the purposes of the FERES initiative, a registry was defined as ‘prospective’ if data collection was pre‐planned and patients were enrolled consecutively at the time of their acute stroke admission or treatment, rather than through retrospective medical chart abstraction. Austria, Greece, Ireland, Italy, and Switzerland were included in this initial proof‐of‐concept phase because they possessed established, high‐quality prospective datasets and were the first partners to successfully complete the legal, ethical, and technical requirements for local Medical Informatics Platform (MIP) node deployment. It is important to note that the capture scale of these registries varies. While some represent nationwide audits of stroke admissions (e.g., INAS in Ireland) or all patients treated within certified stroke unit networks (e.g., SSR in Switzerland, ASUR in Austria), others may represent only those specifically receiving endovascular therapies (e.g., IRETAS in Italy). Consequently, the aggregated data reflects a highly representative, yet selectively enriched, sample of European acute stroke care.

### 2.3 Common Data Elements (CDEs) and Data Harmonization

Cross‐registry analyses were enabled through a stroke‐specific Common Data Element (CDE) model, derived from an established national registry and iteratively expanded during onboarding. CDEs specify standardized names, definitions, data types, permissible values/units, and position along the care pathway (prestroke, admission/baseline, imaging, acute treatment, in‐hospital course, discharge, follow‐up).

A hierarchical design preserved comparability when registries differed in granularity (e.g., mapping detailed categories to harmonized higher‐level variables while retaining local detail where available).

Harmonization followed an ETL process: de‐identified extracts were generated locally, direct identifiers removed, quasi‐identifiers transformed into clinically meaningful intervals (e.g., delays), and variables mapped using registry dictionaries with recoding, unit conversion, and derivation of composite indicators.

Mapping was performed with oversight of a stroke neurologist with expertise in data management. Discrepancies in variable definitions are resolved through structured consultation. Automated checks assessed completeness, plausibility, and internal consistency. Harmonized datasets remained stored locally.

### 2.4 Medical Informatics Platform (MIP) Architecture and Federated Analytics

**FIGURE 1:**
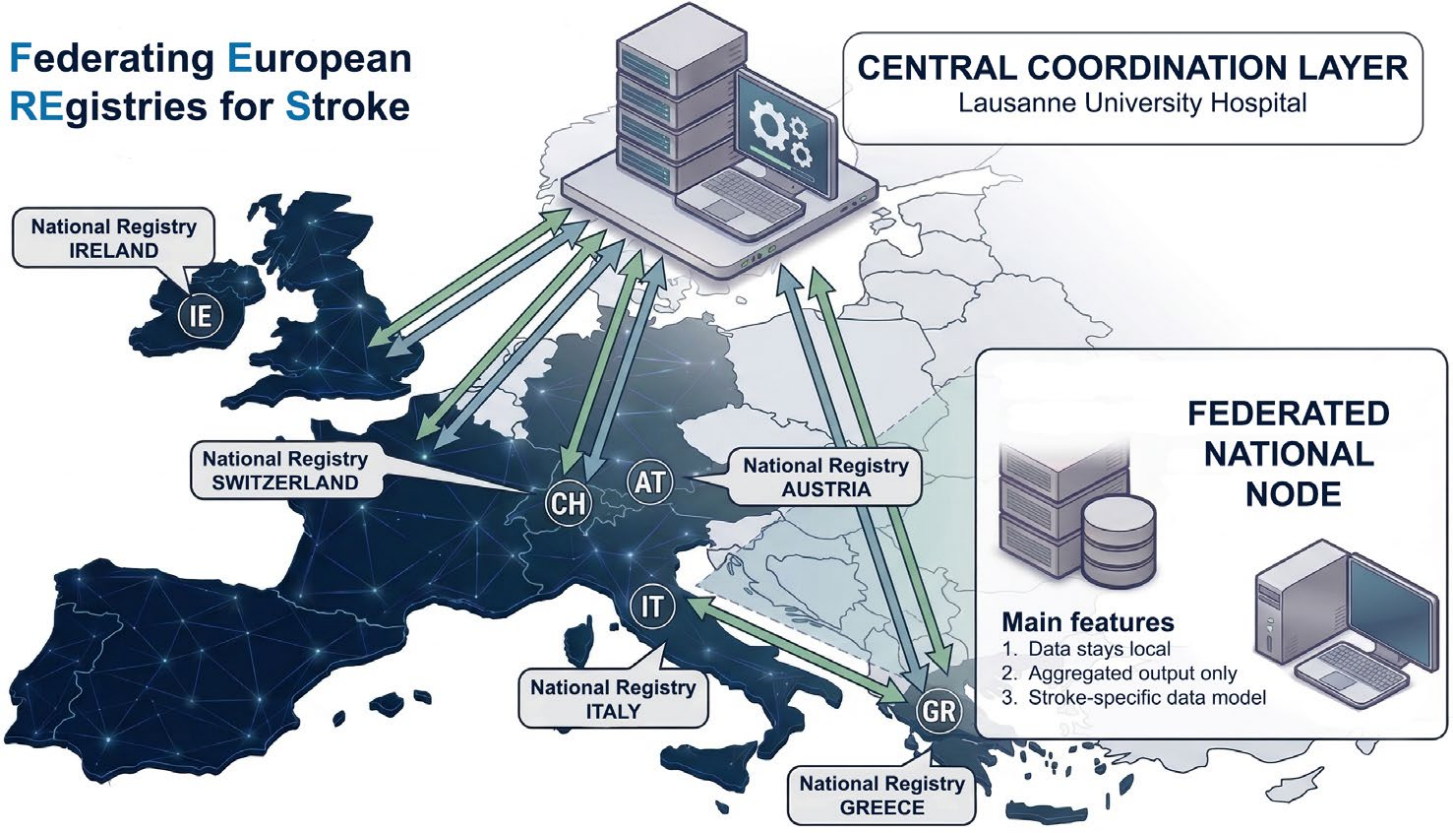
Structure of the federation with central node and local nodes Structure of the federated network including a central coordination layer and one federated node per participating country.

**FIGURE 2:**
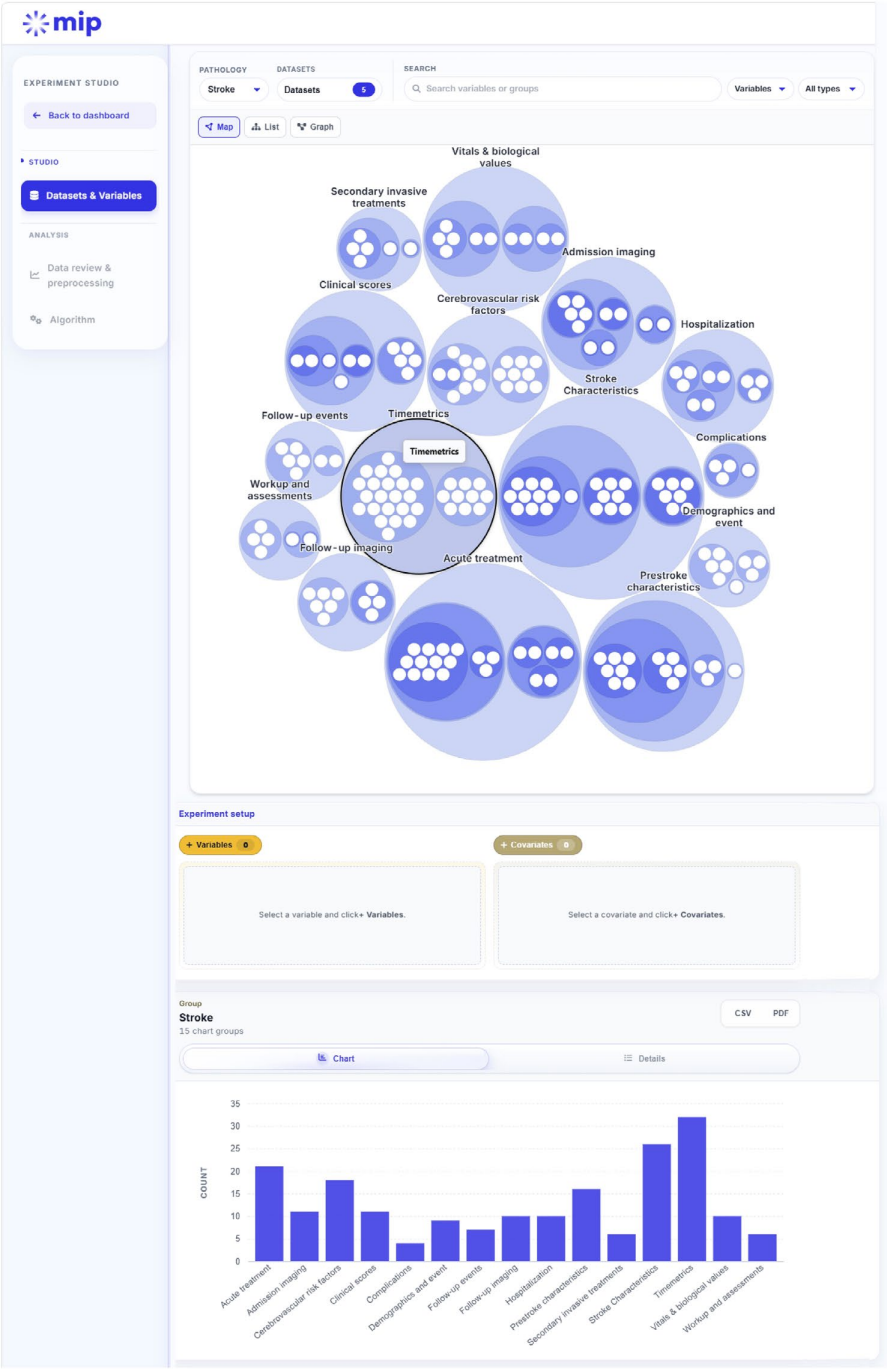
Federated Medical Informatics Platform (MIP) Graphical User Interface in v8.5 This workflow illustrates how a clinical researcher can intuitively select stroke‐specific variables (e.g., NIHSS, age, treatment type) across multiple European registries without requiring direct access to patient‐level data or advanced programming skills. The interface displays the real‐time aggregated results of a decentralized query (e.g., descriptive statistics and regression outputs).

FERES uses the Medical Informatics Platform (MIP) [38], operated within EBRAINS, comprising a central coordination node and multiple local nodes (Figure 1). The central node does not store patient‐level data; it dispatches analysis requests, orchestrates execution, and aggregates summary outputs (e.g., counts, proportions, model coefficients). Analyses follow a “code‐to‐data” paradigm via a web‐based interface (Figure 2) with a predefined method library (descriptive statistics, group comparisons, regression models, and selected machine‐learning methods). For iterative models, multi‐round federated procedures aggregate local updates until convergence. Users cannot access or download individual‐level data; exports are restricted to aggregated, publication‐ready results.

### 2.5 Governance and Security

FERES operates under a formal governance framework defining participation, access, analysis approval, authorship, and publication. Oversight is provided by an Executive Board (legal/data protection/operations) and a Scientific Board (research agenda, CDE maintenance, proposal review). MIP is deployed as containerized services (Kubernetes) with centralized identity and access management, role‐based permissions, continuous monitoring, and full auditability of requests, executions, and exports. Metadata are registered in shared catalogs to support FAIR principles at the metadata level.

### 2.6 Showcase Analysis: Anterior Versus Posterior Circulation Strokes

To validate analytical reproducibility, a predefined showcase compared anterior circulation strokes (ACS) versus posterior circulation strokes (PCS) in a large, harmonized cohort of consecutive acute ischemic stroke admissions (2013–2024). Vascular territory followed registry‐based anatomical definitions mapped to the CDE schema. The protocol specified baseline variables (age, sex, NIHSS), acute treatments (intravenous thrombolysis, endovascular therapy), and 3‐month outcomes (mRS, mortality). The dataset was deployed in federated mode across separate local nodes to emulate a multi‐site configuration, with a pooled fully anonymized centralized version from the Swiss Stroke Registry data as reference for the only purpose of the analytical validation of the infrastructure. Descriptive statistics, group comparisons, and multivariable regression models were run with identical specifications under centralized and federated modes. Concordance was quantified using absolute percentage error for descriptive statistics and absolute deviation of regression coefficients in log‐odds space. Missing data were handled at the level of each predefined analysis. For the showcase comparison, analyses of 3‐month outcomes were performed as complete‐case analyses: patients without available 3‐month mRS or mortality data were excluded only from the corresponding outcome models, whereas they remained eligible for baseline, treatment, and other analyses for which the required variables were available. No imputation was applied in the primary showcase analysis, because its purpose was to validate concordance between centralized and federated execution using identical analytical specifications rather than to estimate causal treatment effects. The number of patients contributing to each outcome analysis was reported explicitly, including the availability of 3‐month outcome data in 79,370 of 104,679 patients, corresponding to 75.8% of the analytic cohort.

## 3 Results

### 3.1 Participating Stroke Registries and Datasets

Five national stroke registries have formally joined the initial FERES federation: the Austrian Stroke Unit Registry (ASUR) [39], the Greek data from the Registry of Stroke Care Quality (RES‐Q) [40], the Irish National Audit of Stroke (INAS) [41], the Italian Registry of Endovascular Treatment in Acute Stroke (IRETAS) [42], and the Swiss Stroke Registry (SSR) [43].

At the time of analysis, 149,772 patient events from two registries (Swiss Stroke Registry and Greek data from RES‐Q) were technically accessible for federated querying across active MIP nodes. The remaining three registries (ASUR, INAS, and IRETAS) were technically integrated and in advanced onboarding stages waiting for the administrative process.

The SSR contributed 145,147 admissions (January 2013–March 2025) across 26 hospitals, including 15,667 from the most recent 12 months. Greek RES‐Q data contributed 4844 admissions (January 2021–March 2025) from 14 hospitals, including 1993 in the past year.

### 3.2 Common Data Elements (CDEs) and Data Harmonization Results

The harmonized FERES ontology (CDE version 37) comprises 945 standardized variables organized within a hierarchical stroke‐specific architecture spanning the full care pathway (Supporting Information).

The structural distribution of variables reflects a balanced representation of clinical domains. The largest domains include admission imaging (188 variables, 19.9%), acute treatment (113 variables, 12.0%), and clinical scores (95 variables, 10.1%). Additional domains include workup and assessments (83 variables, 8.8%), complications (76 variables, 8.0%), stroke characteristics (76 variables, 8.0%), hospitalization (52 variables, 5.5%), time metrics (52 variables, 5.5%), prestroke characteristics (49 variables, 5.2%), and cerebrovascular risk factors (45 variables, 4.8%). Follow‐up imaging (33 variables, 3.5%), vital and biological parameters (25 variables, 2.7%), demographics and index event descriptors (23 variables, 2.4%), secondary invasive treatments (18 variables, 1.9%), and follow‐up events (16 variables, 1.7%) complete the ontology structure. The hierarchical structure enables preservation of registry‐specific granularity while ensuring cross‐registry comparability.

### 3.3 Showcase Analysis Protocol: Anterior Versus Posterior Circulation Strokes

From the harmonized extract (145,147 admissions), applying eligibility criteria (AIS, admission ≤ 24 h from last known well, age > 20) yielded an analytic cohort of 104,679 patients. To emulate federation, this cohort was partitioned into two comparable shards: Node‐0 (52,929 admissions) and Node‐1 (51,750 admissions), loaded on separate MIP nodes. Of these, 74,183 (70.9%) were classified as anterior circulation strokes (ACS) and 30,496 (29.1%) as posterior circulation strokes (PCS).

### 3.4 Baseline Characteristics

In the centralized analyses, overall median age was 75 (IQR 64–83). PCS patients were younger than ACS patients (73 [IQR 61–81] vs. 76 [IQR 65–83]). Median admission NIHSS was 2 (IQR 1–6) overall, higher in ACS than PCS (3 (IQR 1–8) vs. 2 (IQR 0–4)) (Table 1). Intravenous thrombolysis was administered in 27.8% of ACS and 17.3% of PCS patients (χ^2^ *p* < 0.05; Cramér's V = 0.10) (Table 2 and Table 4), and endovascular therapy in 18.7% of ACS and 6% of PCS cases (χ^2^ *p* < 0.05; Cramér's V = 0.14) (Table 2 and Table 4), reflecting modest but statistically significant differences in treatment distribution between ACS and PCS. Three‐month outcome data were available in 79,370/104,679 (75.8%) patients. Favorable functional outcome (mRS 0–2) occurred in 74.1% overall, corresponding to 71.6% in ACS and 80.2% in PCS (absolute difference 8.6%, 95% CI 8%–9.2%; *p* < 0.001).

**TABLE 1:** Absolute percentage error (APE) by method versus centralized for admission NIHSS, NIHSS 24 h, age, IVT, EVT, sex (female).

Summary type	Variable	Descriptive metric	Group	Centralized value	Federated value	Node‐0 value	Node‐1 value	APE federated (%)	APE node‐0 (%)	APE node‐1 (%)
Median	Age	Age Median	ACS	76.00	75.81	75.00	76.00	0.25	1.32	0.00
Median	Age	Age Median	PCS	73.00	72.89	72.00	74.00	0.16	1.37	1.37
Median	Age	Age Median	Overall	75.00	74.85	74.00	75.00	0.20	1.33	0.00
Median	NIHSS admission	Admission NIHSS Median	ACS	3.00	2.97	3.00	3.00	1.06	0.00	0.00
Median	NIHSS admission	Admission NIHSS Median	PCS	2.00	1.94	2.00	2.00	3.19	0.00	0.00
Median	NIHSS admission	Admission NIHSS Median	Overall	2.00	2.07	3.00	2.00	3.74	50.00	0.00
Median	NIHSS 24 h	NIHSS 24 h Median	ACS	2.00	1.99	2.00	2.00	0.69	0.00	0.00
Median	NIHSS 24 h	NIHSS 24 h Median	PCS	1.00	1.02	1.00	1.00	2.46	0.00	0.00
Median	NIHSS 24 h	NIHSS 24 h Median	Overall	1.00	1.12	1.00	1.00	11.77	0.00	0.00
Mean	Age	Age Mean	ACS	73.13	73.13	72.72	73.55	0.00	0.56	0.58
Mean	Age	Age Mean	PCS	70.30	70.30	69.44	71.13	0.00	1.21	1.18
Mean	Age	Age Mean	Overall	72.30	72.30	71.79	72.82	0.00	0.71	0.72
Mean	NIHSS 24 h	NIHSS 24 h Mean	ACS	4.33	4.33	4.50	4.13	0.00	4.06	4.55
Mean	NIHSS 24 h	NIHSS 24 h Mean	PCS	2.40	2.40	2.42	2.37	0.00	0.97	1.01
Mean	NIHSS 24 h	NIHSS 24 h Mean	Overall	3.78	3.78	3.93	3.62	0.00	3.93	4.31
Mean	EVT	EVT Mean (%)	ACS	18.67	18.67	18.19	19.18	0.00	2.57	2.72
Mean	EVT	EVT Mean (%)	PCS	6.00	6.00	6.19	5.82	0.00	3.09	3.07
Mean	EVT	EVT Mean (%)	Overall	15.04	15.04	14.83	15.27	0.00	1.43	1.48
Mean	IVT	IVT Mean (%)	ACS	27.81	27.81	27.64	27.99	0.00	0.61	0.64
Mean	IVT	IVT Mean (%)	PCS	17.28	17.28	15.99	18.57	0.00	7.49	7.44
Mean	IVT	IVT Mean (%)	Overall	24.80	24.80	24.38	25.23	0.00	1.69	1.76
Mean	Female	Female Mean (%)	ACS	44.98	44.98	44.79	45.19	0.00	0.44	0.45
Mean	Female	Female Mean (%)	PCS	40.12	40.12	39.64	40.58	0.00	1.20	1.16
Mean	Female	Female Mean (%)	Overall	43.56	43.56	43.33	43.81	0.00	0.55	0.56
Std	Age	Age Standard Deviation	ACS	13.52	13.52	13.60	13.42	0.00	0.61	0.74
Std	Age	Age Standard Deviation	PCS	14.08	14.08	14.23	13.89	0.00	1.03	1.36
Std	Age	Age Standard Deviation	Overall	13.74	13.74	13.86	13.61	0.00	0.83	1.01
Std	NIHSS 24 h	NIHSS 24 h Standard Deviation	ACS	6.62	6.62	6.82	6.39	0.00	2.93	3.47
Std	NIHSS 24 h	NIHSS 24 h Standard Deviation	PCS	4.89	4.89	4.98	4.81	0.00	1.73	1.83
Std	NIHSS 24 h	NIHSS 24 h Standard Deviation	Overall	6.24	6.24	6.43	6.03	0.00	3.01	3.47

**TABLE 2:** Absolute differences for counts, proportions, and unit errors for means/SDs by method versus centralized.

Descriptive metric	Centralized value	Federated value	Node‐0 value	Node‐1 value	Absolute error		
					Federated	Node‐0	Node‐1
Count (ACS)	74183.000	74183.000	37919.000	36264.000	0.000	36264.000	37919.000
Count (Overall)	104679.000	104679.000	52929.000	51750.000	0.000	51750.000	52929.000
Count (PCS)	30496.000	30496.000	15010.000	15486.000	0.000	15486.000	15010.000
Age Mean (ACS)	73.125	73.125	72.719	73.550	0.000	0.406	0.425
Age Mean (Overall)	72.302	72.302	71.791	72.825	0.000	0.511	0.523
Age Mean (PCS)	70.297	70.297	69.444	71.126	0.000	0.854	0.828
NIHSS 24 h Mean (ACS)	4.329	4.329	4.504	4.132	0.000	0.176	0.197
NIHSS 24 h Mean (Overall)	3.780	3.780	3.929	3.618	0.000	0.149	0.163
NIHSS 24 h Mean (PCS)	2.398	2.398	2.422	2.374	0.000	0.023	0.024
EVT Rate (%) (ACS)	18.670	18.670	18.189	19.178	0.000	0.480	0.508
EVT Rate (%) (Overall)	15.044	15.044	14.829	15.267	0.000	0.215	0.223
EVT Rate (%) (PCS)	6.005	6.005	6.190	5.820	0.000	0.185	0.184
IVT Rate (%) (ACS)	27.807	27.807	27.638	27.986	0.000	0.169	0.179
IVT Rate (%) (Overall)	24.795	24.795	24.376	25.230	0.000	0.419	0.435
IVT Rate (%) (PCS)	17.282	17.282	15.987	18.568	0.000	1.295	1.286
Male Percentage (ACS)	55.019	55.019	55.215	54.814	0.000	0.196	0.205
Male Percentage (Overall)	56.435	56.435	56.674	56.191	0.000	0.239	0.244
Male Percentage (PCS)	59.882	59.882	60.363	59.416	0.000	0.482	0.466
Age Standard Deviation (ACS)	13.519	13.519	13.601	13.419	0.000	0.082	0.099
Age Standard Deviation (Overall)	13.745	13.745	13.859	13.607	0.000	0.115	0.138
Age Standard Deviation (PCS)	14.080	14.080	14.225	13.888	0.000	0.145	0.192
NIHSS 24 h Standard Deviation (ACS)	6.623	6.623	6.817	6.393	0.000	0.194	0.230
NIHSS 24 h Standard Deviation (Overall)	6.243	6.243	6.430	6.026	0.000	0.188	0.217
NIHSS 24 h Standard Deviation (PCS)	4.894	4.894	4.979	4.805	0.000	0.085	0.089

### 3.5 Unadjusted and Adjusted Outcome Analyses

In unadjusted logistic regression, PCS compared with ACS was associated with an odds ratio (OR) for favorable outcome of 1.61 (95% CI 1.55–1.67; *p* < 0.001) and an OR for mortality of 0.51 (95% CI 0.47–0.55; *p* < 0.001). After adjustment for age, sex, baseline NIHSS at admission, intravenous thrombolysis, and endovascular therapy, the adjusted OR for favorable outcome was 1.01 (95% CI 0.96–1.06; *p* < 0.001), and for mortality 0.8 (95% CI 0.74–0.87; *p* < 0.001).

### 3.6 Concordance Between Centralized and Federated Analyses

Federated execution reproduced centralized analyses without measurable numerical deviation. Sample counts were identical across execution modes (ACS: 74,183; PCS: 30,496; total: 104,679). Means and standard deviations for age were numerically indistinguishable (Absolute Percentage Error = 0.00%), with identical values for ACS (73.13 years), PCS (70.3 years), and the overall cohort (72.3 years).

All ACS–PCS hypothesis tests yielded identical statistical decisions under centralized and federated execution. *p*‐values for comparisons of age, admission NIHSS, sex distribution, intravenous thrombolysis, and endovascular therapy were identical to displayed precision (Table 3).

**TABLE 3:** Concordance of ACS–PCS hypothesis‐test decisions across methods.

Descriptive metric	Centralized value	Federated value	Node‐0 value	Node‐1 value	Federated	Node‐0	Node‐1
Chi‐Square *p*‐value – EVT (ACS vs. PCS)	0	0	0	0	TRUE (*p* < 0.05)	TRUE (*p* < 0.05)	TRUE (*p* < 0.05)
Chi‐Square *p*‐value – IVT (ACS vs. PCS)	0	0	0	0	TRUE (*p* < 0.05)	TRUE (*p* < 0.05)	TRUE (*p* < 0.05)
Chi‐Square *p*‐value—Sex (ACS vs. PCS)	0	0	0	0	TRUE (*p* < 0.05)	TRUE (*p* < 0.05)	TRUE (*p* < 0.05)
Mann–Whitney *p*‐value – Age (ACS vs. PCS)	0	0	0	0	TRUE (*p* < 0.05)	TRUE (*p* < 0.05)	TRUE (*p* < 0.05)
Mann–Whitney *p*‐value – NIHSS Admission (ACS vs. PCS)	0	0	0	0	TRUE (*p* < 0.05)	TRUE (*p* < 0.05)	TRUE (*p* < 0.05)
*t*‐test *p*‐value – Age (ACS vs. PCS)	0	0	0	0	TRUE (*p* < 0.05)	TRUE (*p* < 0.05)	TRUE (*p* < 0.05)
*t*‐test *p*‐value – NIHSS Admission (ACS vs. PCS)	0	0	0	0	TRUE (*p* < 0.05)	TRUE (*p* < 0.05)	TRUE (*p* < 0.05)

Effect sizes were preserved across execution modes. Cohen's d for admission NIHSS was 0.39 in both analyses; rank‐biserial correlation for admission NIHSS was −0.21 versus −0.20; rank‐biserial correlation for age was −0.11 in both; and Cramér's V for EVT was 0.14 in both (Table 4).

**TABLE 4:** *Effect sizes (Cohen's d, rank‐biserial r, Cramér's V) for ACS* vs *PCS*.

Measure ACS vs PCS	Effect size	Centralized	Federated	On Node_0	On Node_1
NIHSS at admission (t‐test)	Cohen's d	0.39	0.39	0.40	0.37
NIHSS at admission (Mann–Whitney)	Rank‐biserial r	−0.21	−0.20	−0.20	−0.21
Age (Mann–Whitney)	Rank‐biserial r	−0.11	−0.11	−0.13	−0.09
EVT (Chi‐square)	Cramér's V	0.14	0.14	0.14	0.15
IVT (Chi‐square)	Cramér's V	0.10	0.10	0.11	0.09
Sex (Chi‐square)	Cramér's V	0.04	0.04	0.05	0.04

Multivariable regression coefficients were numerically equivalent between centralized and federated models. For the adjusted model predicting favorable outcome, the centralized adjusted OR was 0.98 (95% CI 0.94–1.03) and the federated adjusted OR was 0.98 (95% CI 0.94–1.03). The absolute deviation score in log‐odds space was 0.29 for federated execution, compared with 2.46 and 2.11 for individual real‐data shards (Table 5).

**TABLE 5:** Log‐OR score summarizing absolute differences of regression coefficients in log‐odds space versus centralized.

Descriptive metric	Federated	Node‐0	Node‐1
Outcome Model	0.29	2.46	2.11

Taken together, federated execution reproduced pooled‐data descriptive statistics (Table 1 and Table 2), hypothesis tests (Table 3), effect sizes (Table 4), and multivariable regression coefficients (Table 5) with only minimal numerical discrepancies (e.g., minor fractional rounding differences inherent to decentralized computation)while maintaining full local data governance.

## 4 Discussion

FERES demonstrates the feasibility of conducting large‐scale, multinational stroke research in Europe using a federated, GDPR‐compliant framework. By enabling analyses across national registries without transferring patient‐level data, FERES addresses a core limitation of the current European landscape: fragmentation of real‐world evidence despite the presence of high‐quality registries. Importantly, federation is not merely a regulatory solution; it is a scientifically robust alternative to traditional data pooling.

Furthermore, FERES represents a potential significant step toward fulfilling the monitoring and quality improvement objectives outlined in the Stroke Action Plan for Europe (SAP‐E) 2018–2030 [24]. By establishing a privacy‐preserving, federated infrastructure, FERES directly addresses the SAP‐E's call for systematic, multinational evaluation of stroke care delivery and benchmarking. Through the harmonization of heterogeneous registries into a unified Common Data Model, FERES provides the necessary tools to monitor progress toward key SAP‐E targets, such as reducing geographic inequities in access to reperfusion therapies and specialized stroke unit care. The need for such infrastructure is underscored by persistent and substantial disparities in access to evidence‐based stroke care across Europe. Recent multinational data demonstrate marked heterogeneity in endovascular thrombectomy (EVT) availability and utilization: although 15%–20% of acute ischemic stroke patients may be eligible for EVT, adoption rates range from less than 1% to 7% across different regions [17] and a European survey estimated that only 1.9% of all stroke patients undergo mechanical thrombectomy [21]. A global survey across 89 countries identified cost, late presentation, and shortage of trained neurointerventionalists as the most common barriers to EVT delivery, with 22% of respondents reporting no available thrombectomy service [19]. These infrastructure gaps are compounded by geographic and socioeconomic disparities: rural populations have significantly lower odds of receiving EVT compared with urban populations (OR 0.39, *p* < 0.001), and patients with low socioeconomic status face similarly reduced access (OR 0.74, *p* < 0.001) [15]. Importantly, these inequalities persist even within countries with universal tax‐funded healthcare systems [18], suggesting that structural barriers extend beyond insurance coverage alone. A systematic review and modified Delphi consensus identified 12 key barriers to mechanical thrombectomy access globally, including public awareness, emergency medical services infrastructure, prehospital screening protocols, interhospital transfer policies, stroke imaging availability, neurointerventional expertise, and device availability [44]. Furthermore, a recent cost‐effectiveness analysis across 32 European countries demonstrated that mechanical thrombectomy is cost‐effective in virtually all settings, yet implementation remains far below optimal levels [20]. Through the harmonization of heterogeneous registries into a unified Common Data Model, FERES provides the necessary tools to monitor progress toward key SAP‐E targets, such as reducing these geographic and socioeconomic inequities in access to reperfusion therapies and specialized stroke unit care.

A key contribution is the empirical validation of federated analytics. In a predefined showcase analysis, federated execution reproduced pooled centralized results across descriptive statistics, hypothesis testing, effect‐size estimation, and regression modeling, without measurable numerical drift. This analytical fidelity is critical for clinician and stakeholder trust, showing that privacy‐preserving computation can deliver evidence comparable to conventional centralized workflows while keeping data in situ.

While the initial showcase analyses and interface illustrations focus predominantly on acute‐phase metrics, the comprehensive FERES Common Data Elements (CDE) ontology is designed to span the entire stroke care continuum. Crucially, this includes the harmonized capture of the post‐hospital pathway, incorporating variables such as specific discharge destinations, rehabilitation settings, and long‐term functional follow‐up. An exhaustive list of all variables currently integrated into the FERES CDE schema at the time of publication is available in the Supporting Information.

The scientific value of FERES lies in scale and heterogeneity. Individual national registries remain essential for quality monitoring and local research but are often underpowered for modest effects, rare outcomes, and infrequent subgroups. Federation increases effective sample size and geographic representativeness, improving precision and generalizability across diverse healthcare systems—an especially relevant advantage in stroke, where care pathways and access to therapies vary substantially and influence outcomes. This is particularly important for understanding patterns of EVT underutilization, which exist in virtually any healthcare system and range from complete lack of access to selective undertreatment of certain patient subgroups [45]. By aggregating data across countries with different levels of EVT infrastructure and implementation, FERES can enable comparative effectiveness research, identify system‐level bottlenecks, and support targeted quality improvement interventions tailored to specific healthcare contexts.

Interoperability is another major advance. The stroke‐specific Common Data Elements (CDE) model provides a standardized semantic layer that preserves clinical meaning while enabling consistent cross‐registry analyses. Its hierarchical structure accommodates differences in granularity across registries, supporting comparability without forcing information loss. By embedding harmonization within the analytical infrastructure, FERES shifts from ad hoc mapping toward a reusable platform that can support successive studies as additional registries join.

FERES also improves research efficiency. Once onboarding and mapping are completed, approved analyses can be executed rapidly without repeated data‐transfer negotiations or bespoke harmonization, lowering barriers to replication and multicountry validation.

Beyond research, FERES has direct implications for quality improvement and health policy. Federated analyses of key performance indicators, treatment rates, workflow delays, and outcomes enable benchmarking across institutions and countries while respecting national data governance, supporting the Stroke Action Plan for Europe and similar initiatives aimed at reducing unwarranted variation in care. The capacity to conduct cross‐national comparisons while maintaining local data sovereignty is particularly valuable for addressing the documented variations in quality indicators across European countries, including substantial differences in stroke unit admission rates (12–100%), door‐to‐imaging times (7–41 min), door‐to‐needle times (20–75 min), and intravenous thrombolysis rates (< 1% to 52%) [13]. Such benchmarking can identify best practices, reveal modifiable system‐level factors contributing to disparities, and inform evidence‐based policy interventions to improve equitable access to time‐sensitive stroke treatments. Importantly, FERES complements randomized controlled trials by providing real‐world evidence on effectiveness, implementation, and long‐term outcomes in unselected populations.

Several limitations should be acknowledged. Federated analysis cannot fully replace direct access to patient‐level data in all scenarios. Highly customized modeling strategies, complex data cleaning procedures, or advanced imputation techniques may be more challenging in a distributed setting. As with all registry‐based research, results depend on the completeness and quality of source data, which harmonization cannot entirely correct. Implementation remains resource intensive. Establishing federated participation required alignment across institutional leadership, ethics/legal teams, data protection officers, and local IT, including explicit delineation of controller–processor roles under GDPR and validation of ETL processes. These steps are demanding but foundational for long‐term trust and sustainability.

Future development should focus on expanding registry participation to increase geographic representation and statistical power, particularly including registries from countries with lower EVT availability and different healthcare system structures to enable more comprehensive assessment of access disparities and their determinants. Enriching the analytical library with additional statistical methods commonly used in stroke research—such as time‐to‐event models, competing‐risk analyses, causal inference techniques, and federated machine learning algorithms—will further enhance scientific scope. Integration of additional data modalities, including imaging repositories and administrative health records, represents an important next step toward multimodal federated stroke analytics. Streamlining onboarding procedures and enabling near‐real‐time monitoring of key performance indicators would strengthen the platform's role in continuous quality improvement. Furthermore, FERES could serve as a model for implementation research studies examining system‐level interventions to reduce EVT underutilization, such as optimizing interhospital transfer protocols, expanding telestroke networks, or deploying mobile stroke units in underserved regions [46].

Overall, FERES provides a scalable and trustworthy framework for collaborative stroke research in Europe, aligning clinically meaningful harmonization, robust governance, and privacy‐preserving analytics to enable cross‐border real‐world evidence generation that can directly inform efforts to reduce persistent inequalities in access to evidence‐based stroke care.

## 5 Conclusions

FERES demonstrates that large‐scale, multinational stroke research in Europe is achievable through a federated, privacy‐preserving approach. By combining a harmonized stroke‐specific CDE model, validated federated analytics, and structured governance, FERES enables secure cross‐border reuse of registry data without centralizing patient‐level information. Federated execution reproduced centralized results without numerical drift, confirming that privacy‐preserving computation can maintain analytical rigor.

By linking national registries within a shared infrastructure, FERES increases scale, improves external validity, and enables analyses of rare subgroups and heterogeneous care pathways that are difficult to study within isolated datasets. As European initiatives increasingly promote secure secondary use of health data, FERES offers a clinically grounded and scalable solution to support real‐world evidence generation, international benchmarking, and data‐driven quality improvement in stroke care.

## Author Contributions

**Danilo Toni:** writing – review and editing, project administration, resources, supervision, data curation. **Michael Knoflach:** resources, data curation, project administration, writing – review and editing. **Mira Katan:** data curation, writing – review and editing, project administration, resources. **Leonardo Renieri:** writing – review and editing, project administration, resources, data curation. **Stefan Kiechl:** writing – review and editing, resources, project administration, data curation. **Joseph Harbison:** data curation, resources, project administration, writing – review and editing. **Andreas Ktenidis:** formal analysis, writing – review and editing, writing – original draft, methodology. **Alexander Salerno:** conceptualization, methodology, formal analysis, data curation, writing – original draft, writing – review and editing, visualization, validation, project administration, investigation. **Theofilos Mailis:** writing – review and editing, formal analysis, methodology, writing – original draft. **Georges Melissargos:** conceptualization, methodology, data curation, formal analysis, validation, visualization, writing – review and editing, project administration. **Yannis Ioannidis:** writing – review and editing, formal analysis, methodology, writing – original draft. **Lina Palaiodimou:** data curation, resources, project administration, writing – review and editing. **Philippe Ryvlin:** funding acquisition, writing – review and editing, project administration, resources, supervision, conceptualization, methodology. **Patrik Michel:** conceptualization, supervision, project administration, resources, writing – review and editing, funding acquisition, methodology. **Georgios Tsivigoulis:** writing – review and editing, project administration, resources, data curation.

## Funding

This publication is based on research and development work carried out within the FERES Project. The authors acknowledge EAN as partner in the project. This work was supported by additional funding provided by EAN. The publication of these results was approved in advance by EAN. Part of the work for FERES is co‐funded by the Horizon Europe R&I program through the EBRAINS2.0 project (101147319) and SERI (23.00638). The MIP is a service provided by the EBRAINS Research Infrastructure. The European Stroke Organization (ESO) provides non‐financial support to the initiative.

## Conflicts of Interest

The authors declare no conflicts of interest.

## Supporting information


**Data S1:** Common Data Elements variables (version 37).

## Data Availability

The raw, anonymized data that support the findings of this study are available from the corresponding author upon reasonable request and after signing a data transfer and use agreement. If such data are used for a publication, its methods should be communicated, and internationally recognized authorship rules should be applied.

## References

[1] Collaborators GBDSRF , “Global, Regional, and National Burden of Stroke and Its Risk Factors, 1990‐2021: A Systematic Analysis for the Global Burden of Disease Study 2021,” Lancet Neurology 23, no. 10 (2024): 973–1003. 39304265 10.1016/S1474-4422(24)00369-7 PMC12254192

[2] L. Pu , L. Wang , R. Zhang , T. Zhao , Y. Jiang , and L. Han , “Projected Global Trends in Ischemic Stroke Incidence, Deaths and Disability‐Adjusted Life Years From 2020 to 2030,” Stroke 54, no. 5 (2023): 1330–1339. 37094034 10.1161/STROKEAHA.122.040073

[3] S. Man , N. Solomon , B. Mac Grory , et al., “Shorter Door‐To‐Needle Times Are Associated With Better Outcomes After Intravenous Thrombolytic Therapy and Endovascular Thrombectomy for Acute Ischemic Stroke,” Circulation 148, no. 1 (2023): 20–34. 37199147 10.1161/CIRCULATIONAHA.123.064053 PMC10356148

[4] E. Berge , W. Whiteley , H. Audebert , et al., “European Stroke Organisation (ESO) Guidelines on Intravenous Thrombolysis for Acute Ischaemic Stroke,” European Stroke Journal 6, no. 1 (2021): I–LXII. 10.1177/2396987321989865 PMC7995316 33817340

[5] G. Turc , P. Bhogal , U. Fischer , et al., “European Stroke Organisation (ESO) ‐ European Society for Minimally Invasive Neurological Therapy (ESMINT) Guidelines on Mechanical Thrombectomy in Acute Ischemic Stroke,” Journal of NeuroInterventional Surgery 15, no. 8 (2023): e8. 30808653 10.1136/neurintsurg-2018-014569

[6] J. P. Marto , A. Salerno , E. Maslias , et al., “Stroke in the Stroke Unit: Recognition, Treatment and Outcomes in a Single‐Center Cohort,” European Journal of Neurology 29 (2022): 2674–2682. 35608958 10.1111/ene.15415

[7] D. Leys , “Another Benefit of Stroke Unit Care,” European Journal of Neurology 29 (2022): 2557–2558. 35767393 10.1111/ene.15480

[8] P. Langhorne , S. Ramachandra , and Stroke Unit Trialists C , “Organised Inpatient (Stroke Unit) Care for Stroke: Network Meta‐Analysis,” Cochrane Database of Systematic Reviews 4, no. 4 (2020): CD000197. 32324916 10.1002/14651858.CD000197.pub4 PMC7197653

[9] N. A. Hilkens , B. Casolla , T. W. Leung , and F. E. de Leeuw , “Stroke,” Lancet 403, no. 10446 (2024): 2820–2836. 38759664 10.1016/S0140-6736(24)00642-1

[10] T. L. Green , N. D. McNair , J. L. Hinkle , et al., “Care of the Patient With Acute Ischemic Stroke (Posthyperacute and Prehospital Discharge): Update to 2009 Comprehensive Nursing Care Scientific Statement: A Scientific Statement From the American Heart Association,” Stroke 52, no. 5 (2021): e179–e197. 33691469 10.1161/STR.0000000000000357

[11] P. Langhorne , H. J. Audebert , D. A. Cadilhac , J. Kim , and P. Lindsay , “Stroke Systems of Care in High‐Income Countries: What Is Optimal?,” Lancet 396, no. 10260 (2020): 1433–1442. 33129394 10.1016/S0140-6736(20)31363-5

[12] U. Fischer , Inequity in Access to Stroke Care in Europe: Overlook Based on Mappings (European Stroke Organisation Conference, 2022).

[13] R. Mikulik , G. Neto , R. Sedani , et al., “Differences in Acute Ischemic Stroke Treatment: A Cross‐Sectional Study From International Registry of Stroke Care Quality (RES‐Q),” International Journal of Stroke 21, no. 2 (2026): 188–199. 40698920 10.1177/17474930251364082

[14] Z. Luo , D. Jiang , S. Shan , et al., “Cross‐Country Inequalities in Disease Burden and Quality of Care of Stroke, 1990‐2021: A Systematic Analysis of the Global Burden of Disease Study 2021,” European Journal of Neurology 32, no. 2 (2025): e70050. 39878377 10.1111/ene.70050 PMC11775921

[15] R. Biswas , T. Wijeratne , K. Zelenak , et al., “Disparities in Access to Reperfusion Therapy for Acute Ischemic Stroke (DARTS): A Comprehensive Meta‐Analysis of Ethnicity, Socioeconomic Status, and Geographical Factors,” CNS Drugs 39, no. 4 (2025): 417–442. 39954118 10.1007/s40263-025-01161-z

[16] P. Langhorne , M. J. O'Donnell , S. L. Chin , et al., “Practice Patterns and Outcomes After Stroke Across Countries at Different Economic Levels (INTERSTROKE): An International Observational Study,” Lancet 391, no. 10134 (2018): 2019–2027. 29864018 10.1016/S0140-6736(18)30802-X

[17] D. Lauer , J. Sulzenko , H. Malikova , I. Stetkarova , and P. Widimsky , “Advances in Endovascular Thrombectomy for the Treatment of Acute Ischemic Stroke,” Expert Review of Neurotherapeutics 25, no. 6 (2025): 675–687. 40200903 10.1080/14737175.2025.2490538

[18] S. M. O. Buus , M. L. Schmitz , P. Cordsen , S. P. Johnsen , G. Andersen , and C. Z. Simonsen , “Socioeconomic Inequalities in Reperfusion Therapy for Acute Ischemic Stroke,” Stroke 53, no. 7 (2022): 2307–2316. 35579017 10.1161/STROKEAHA.121.037687

[19] A. Nasreldein , W. Asyraf , T. N. Nguyen , et al., “Global Challenges in the Access of Endovascular Treatment for Acute Ischemic Stroke (Global MT Access),” International Journal of Stroke 20, no. 6 (2025): 660–668. 39773209 10.1177/17474930251314395

[20] P. Candio , M. Violato , J. Leal , and R. Luengo‐Fernandez , “Cost‐Effectiveness of Mechanical Thrombectomy for Treatment of Nonminor Ischemic Stroke Across Europe,” Stroke 52, no. 2 (2021): 664–673. 33423511 10.1161/STROKEAHA.120.031027 PMC7834665

[21] D. Aguiar de Sousa , R. von Martial , S. Abilleira , et al., “Access to and Delivery of Acute Ischaemic Stroke Treatments: A Survey of National Scientific Societies and Stroke Experts in 44 European Countries,” European Stroke Journal 4, no. 1 (2019): 13–28. 31165091 10.1177/2396987318786023 PMC6533860

[22] B. Norrving , J. Barrick , A. Davalos , et al., “Action Plan for Stroke in Europe 2018‐2030,” European Stroke Journal 3, no. 4 (2018): 309–336. 31236480 10.1177/2396987318808719 PMC6571507

[23] A. Webb , M. R. Heldner , D. Aguiar de Sousa , et al., “Availability of Secondary Prevention Services After Stroke in Europe: An ESO/SAFE Survey of National Scientific Societies and Stroke Experts,” European Stroke Journal 4, no. 2 (2019): 110–118. 31259259 10.1177/2396987318816136 PMC6572590

[24] H. Christensen , F. R. Pezzella , M. B. Roaldsen , et al., “Stroke Action Plan for Europe 2018–2030 (SAP‐E): Mid‐Term Review and Update,” European Stroke Journal 11, no. 1 (2026): aakaf026. 41614539 10.1093/esj/aakaf026 PMC12866651

[25] S. Wiedmann , B. Norrving , T. Nowe , et al., “Variations in Quality Indicators of Acute Stroke Care in 6 European Countries: The European Implementation Score (EIS) Collaboration,” Stroke 43, no. 2 (2012): 458–463. 22156695 10.1161/STROKEAHA.111.628396

[26] B. Norrving , B. D. Bray , K. Asplund , et al., “Cross‐National Key Performance Measures of the Quality of Acute Stroke Care in Western Europe,” Stroke 46, no. 10 (2015): 2891–2895. 26265128 10.1161/STROKEAHA.115.008811

[27] T. R. Frieden , “Evidence for Health Decision Making ‐ Beyond Randomized, Controlled Trials,” New England Journal of Medicine 377, no. 5 (2017): 465–475. 28767357 10.1056/NEJMra1614394

[28] C. Zoccali and G. Tripepi , “Real‐World Evidence and Observational Studies: Methodological Challenges in Clinical Research,” European Journal of Clinical Investigation 56, no. 1 (2026): e70153. 41358794 10.1111/eci.70153

[29] S. V. Wang , S. Schneeweiss , Initiative R‐D , et al., “Emulation of Randomized Clinical Trials With Nonrandomized Database Analyses: Results of 32 Clinical Trials,” JAMA 329, no. 16 (2023): 1376–1385. 37097356 10.1001/jama.2023.4221 PMC10130954

[30] V. L. Bartlett , S. S. Dhruva , N. D. Shah , P. Ryan , and J. S. Ross , “Feasibility of Using Real‐World Data to Replicate Clinical Trial Evidence,” JAMA Network Open 2, no. 10 (2019): e1912869. 31596493 10.1001/jamanetworkopen.2019.12869 PMC6802419

[31] V. Chico , “The Impact of the General Data Protection Regulation on Health Research,” British Medical Bulletin 128, no. 1 (2018): 109–118. 30445448 10.1093/bmb/ldy038

[32] L. Bertolaccini , P. E. Falcoz , A. Brunelli , et al., “The Significance of General Data Protection Regulation in the Compliant Data Contribution to the European Society of Thoracic Surgeons Database,” European Journal of Cardio‐Thoracic Surgery 64, no. 3 (2023): ezad289. 37589648 10.1093/ejcts/ezad289

[33] A. Brauneck , L. Schmalhorst , M. M. Kazemi Majdabadi , et al., “Federated Machine Learning, Privacy‐Enhancing Technologies, and Data Protection Laws in Medical Research: Scoping Review,” Journal of Medical Internet Research 25 (2023): e41588. 36995759 10.2196/41588 PMC10131784

[34] J. Casaletto , A. Bernier , R. McDougall , and M. S. Cline , “Federated Analysis for Privacy‐Preserving Data Sharing: A Technical and Legal Primer,” Annual Review of Genomics and Human Genetics 24 (2023): 347–368. 10.1146/annurev-genom-110122-084756 PMC10846631 37253596

[35] A. Sadilek , L. Liu , D. Nguyen , et al., “Privacy‐First Health Research With Federated Learning,” NPJ Digital Medicine 4, no. 1 (2021): 132. 34493770 10.1038/s41746-021-00489-2 PMC8423792

[36] K. Filippopolitis , Y. Foufoulas , M. Garofalakis , et al., “MIP: Advanced Data Processing and Analytics for Science and Medicine. Proceedings of the 27th International Conference on Extending Database Technology (EDBT),” (2024).

[37] K. Amunts , J. DeFelipe , C. Pennartz , et al., “Linking Brain Structure, Activity, and Cognitive Function Through Computation,” eNeuro 9, no. 2 (2022): ENEURO.0316–ENEU21.2022. 35217544 10.1523/ENEURO.0316-21.2022 PMC8925650

[38] G. Melissargos , B. Schaffhauser , A. Salerno , et al., “Deploying the Medical Informatics Platform for Cross‐Border Federated Analytics in FERES and eCREAM,” Frontiers in Disaster and Emergency Medicine 4 (2026): 1748193.

[39] M. M. Steiner , M. Brainin , and Austrian Stroke Registry for Acute Stroke U , “The Quality of Acute Stroke Units on a Nation‐Wide Level: The Austrian Stroke Registry for Acute Stroke Units,” European Journal of Neurology 10, no. 4 (2003): 353–360. 12823485 10.1046/j.1468-1331.2003.00609.x

[40] L. Palaiodimou , O. Kargiotis , A. H. Katsanos , et al., “Quality Metrics in the Management of Acute Stroke in Greece During the First 5 Years of Registry of Stroke Care Quality (RES‐Q) Implementation,” European Stroke Journal 8, no. 1 Suppl (2023): 5–15. 36793743 10.1177/23969873221103474 PMC9923128

[41] C. N. Moran , I. Jeffares , J. McCormack , et al., “Enhancing Acute Stroke Care in Ireland: A Scoping Review and Delphi Consensus for the Irish National Audit of Stroke (INAS) Dataset,” BMJ Open 15, no. 11 (2025): e102789. 10.1136/bmjopen-2025-102789 PMC12658548 41271407

[42] S. Mangiafico , G. Pracucci , V. Saia , et al., “The Italian Registry of Endovascular Treatment in Acute Stroke: Rationale, Design and Baseline Features of Patients,” Neurological Sciences 36, no. 6 (2015): 985–993. 25567080 10.1007/s10072-014-2053-5

[43] C. Manno , G. Disanto , G. Bianco , et al., “Outcome of Endovascular Therapy in Stroke With Large Vessel Occlusion and Mild Symptoms,” Neurology 93, no. 17 (2019): e1618–e1626. 31591276 10.1212/WNL.0000000000008362

[44] S. R. Aroor , C. B. Zevallos , K. S. Asif , et al., “Mechanical Thrombectomy Access Score: A Systematic Review and Modified Delphi of Global Barriers to Endovascular Therapy,” Stroke 56, no. 1 (2025): 158–167. 39450508 10.1161/STROKEAHA.124.047805

[45] J. M. Ospel , W. K. Diprose , A. Ganesh , et al., “Challenges to Widespread Implementation of Stroke Thrombectomy,” Stroke 55, no. 8 (2024): 2173–2183. 38979609 10.1161/STROKEAHA.124.045889

[46] B. de Greef , J. H. Youn , H. V. Baviere , et al., “Enhancing Stroke Care in Europe: Cost‐Effectiveness Analysis of Strategies Addressing Gaps in Acute Ischemic Stroke Care,” European Stroke Journal 11, no. 5 (2026): aakag041. 42153761 10.1093/esj/aakag041 PMC13184963

